# Perceptions of Workplace Fairness in the Context of Age and Intersectionality

**DOI:** 10.1093/geroni/igaa057.2332

**Published:** 2020-12-16

**Authors:** Cal Halvorsen, Marcie Pitt-Catsouphes, Indrani Saran

**Affiliations:** 1 Boston College, Chestnut Hill, Massachusetts, United States; 2 Boston College School of Social Work, Chestnut Hill, Massachusetts, United States

## Abstract

While scholars have focused on workplace fairness (often called organizational justice) for quite some time, the context of diversity—in its many forms—has rarely been included in this conversation. This presentation will review concepts related to workplace fairness, describing how the context of diversity may influence perceptions of it. We then will present the results of a recent survey of 609 respondents aged 18 to 70 with a focus on how holding diverse attributes (e.g., age, gender, and their intersectionality) may shape perceptions of workplace fairness and diversity. Overall, we found that the perceptions of workplace fairness and diversity are similar by age and gender, with a few notable differences (e.g., older respondents value interpersonal justice the most, such as their opinions being considered, and younger respondents see workplace diversity the most positively). These results can inform scholarship and discussions on human resource practices and environmental change in organizations.

